# Cerebellar repetitive transcranial magnetic stimulation versus propranolol for essential tremor

**DOI:** 10.1002/brb3.2926

**Published:** 2023-02-17

**Authors:** Yue Lv, Mengran Wang, Juan Yang, Jin Shi, Tingting Xuan, Junmei Zhang, Dandan Du, Jiang Cheng, Haining Li

**Affiliations:** ^1^ Department of Neurology General Hospital of Ningxia Medical University Yinchuan China; ^2^ School of Clinical Medicine Ningxia Medical University Yinchuan China; ^3^ Diagnosis and Treatment Engineering Technology Research Center of Nervous System Diseases of Ningxia Hui Autonomous Region Yinchuan China

**Keywords:** essential tremor (ET), propranolol, repetitive transcranial magnetic stimulation (rTMS)

## Abstract

**Background:**

Propranolol, a nonselective beta‐adrenergic blocker, has long been used as one of the standard treatments for essential tremor (ET). Repetitive transcranial magnetic stimulation (rTMS) has also been used for a long time as a substitution therapy for ET patients.

**Objective:**

The main aim of this study was to evaluate the antitremor effect of 1‐Hz (low‐frequency) cerebellar rTMS and compare it to the use of propranolol in ET patients.

**Methods:**

In this single‐blinded, randomized, controlled pilot study, a total of 38 patients with ET were randomized into two groups. One group (*n* = 20) received 1200 pulses of 1‐Hz rTMS at an intensity of 90% of the resting motor threshold to the bilateral cerebellar region for 10 days. Another group (*n* = 18) received oral propranolol for 30 days. The initial dose was 30 mg/day, which was increased to 60 mg/day after 5 days, then to 90 mg/day on the 11th day, and continued thereafter for 20 days. The Fahn–Tolosa–Marin (FTM) clinical scale was assessed at baseline and at days 5, 10, and 30 to evaluate tremor severity, specific motor tasks, and functional disability.

**Results:**

Low‐frequency rTMS of the cerebellum significantly improved tremor severity, specific motor tasks (writing, spiral drawing, and pouring), and FTM total scores on days 10 and 30. Nevertheless, we found no significant difference in functional disability at any point in time (*p* > .05). There were no statistically significant differences in FTM Part A, Part B, Part C scores and total scores of patients in propranolol group on days 5 and 10 compared with before treatment (*p* > .05). However, FTM total scores and FTM Part A, Part B, and Part C scores were significantly improved for patients when the dose of propranolol was 90 mg/day on day 30. Our study showed that there was no statistically significant difference in the total FTM scores and FTM Part A, Part B, and Part C scores between rTMS and propranolol on days 5, 10, and 30 (*p* > .05).

**Conclusion:**

We conclude that both cerebellar low‐frequency rTMS and propranolol could be effective treatment options for patients with ET, but it is not clear which method is more effective.

## Graphical abstract

This paper is the first study to compare the effects of rTMS and medication for ET patients. This will help healthcare providers and ET patients select the best treatment.

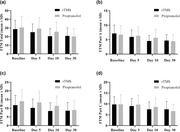

## 1 INTRODUCTION

Essential tremor (ET) is one of the most frequent movement disorders and is defined as 4–12 Hz kinetic or postural tremor mainly involving the bilateral upper region with or without head tremor or tremor in other locations (Bhatia et al., 2018). Although this disorder is not life threatening, tremor usually worsens with age, leading to functional disabilities (i.e., difficulty in writing, pouring liquid with a spoon or glass, and other precise procedures) as well as a loss of independence. ET requires treatment when patients are troubled with the above (Alonso‐Navarro et al., 2020).

Existing studies indicate that propranolol and primidone remain the preferred oral agents for ET, and propranolol can significantly reduce the amplitude of tremor (Schneider & Deuschl, 2014), although there is no correlation between the blood concentration of propranolol and its effectiveness in suppressing tremor (Koller, 1986). Propranolol is considered to be an effective therapy for ET of the upper extremity according to the evidence‐based ET treatment guidelines from the American Academy of Neurology (Zesiewicz et al., 2011), the MDS Task Force on Tremor (Ferreira et al., 2019), and the Italian Movement Disorders Association (Zappia et al., 2013), but it has shown only a slight or no effect on head tremors (Paparella et al., 2018). Various clinical trials and consensus have shown that 50%–70% of ET patients’ symptoms improve with propranolol (Ondo, 2006). However, no clear clinical or physiological factors can accurately predict this response. In general, propranolol can reduce the amplitude of hand tremor by approximately 50% and improve head and voice tremor (Justicz et al., 2016).

As the amplitude of the tremor increased, medication treatment became less effective. Surgical approaches often have been used for drug‐refractory ET, mainly consisting of deep brain stimulation and thalamotomy procedures with radiofrequency, gamma knife radiosurgical thalamotomy, and, most recently, focused ultrasound thalamotomy (Dallapiazza et al., 2019). However, these procedures are invasive, and many patients prefer noninvasive therapeutic options before resorting to surgery. Diverse noninvasive brain stimulations (NIBS), such as repetitive transcranial magnetic stimulation (rTMS), transcranial alternating current stimulation, and theta burst stimulation, have been used as treatments for some ET patients (Kang & Cauraugh, 2017). Repetitive TMS generates a magnetic field near the patient's scalp by stimulating coils that deliver small electrical currents in brain regions. High‐frequency currents (>5 Hz) of rTMS give rise to increased cortical excitability, while low‐frequency currents (≤1 Hz) of rTMS result in cortical inhibition of the target brain region (Lefaucheur et al., 2014). Tremor was associated with pathological oscillations that interpose the function of the cerebellum. In this framework, in addition to originating in the cerebellar cortex, oscillatory activity also occurs throughout the motor network by cerebellar–thalamic–cortex (CTC) pathways (Raethjen & Deuschl, 2012). Based on the participation of CTC in ET occurrence, cerebellar stimulation may be effective in treating patients with ET (Filip et al., 2016). Popa et al. (2013) reported that cerebellar rTMS significantly reduced tremor amplitude and functional disability and improved specific motor tasks (tremor, drawing, and pouring) and total scores. These effects lasted for 3 weeks after the last treatment.

However, existing studies did not compare the efficacy of propranolol and rTMS for ET patients. We present a prospective study to further evaluate the efficacy of propranolol (oral medication) compared with cerebellar low‐frequency rTMS in patients with ET. This will help healthcare providers and ET patients select the best treatment.

## 2 METHODS

### 2.1 Participants

Thirty‐eight patients with ET participated in the study. They were all recruited from the Department of Neurology in the General Hospital of Ningxia Medical University (Yinchuan, Ningxia, China). These patients were diagnosed based on Movement Disorder Society (MDS) criteria (Bhatia et al., 2018). Patients were excluded if they had contraindications to beta‐adrenergic blocker treatment, including bradycardia, hypotension, uncontrolled asthma, or diabetes, or were deemed unable to comply with the research. Patients with a cardiac pacemaker, insulin pump, and other metal devices in their bodies or who had any contraindications for exposure to the magnetic field as well as those with a history of seizure were excluded. ET patients with other neurological signs of uncertain significance such as impaired tandem gait, questionable dystonic posturing, memory impairment, or other mild neurologic signs of unknown significance, also called ET‐Plus (Bhatia et al., 2018), had been exclude. All forms of antitremor drugs were discontinued at least 1 month prior to treatment to achieve stable clinical status.

All participants signed written consent in advance of the intervention. All protocols for this study were approved by the Institutional Review Board of the General Hospital of Ningxia Medical University.

### 2.2 Procedures

Subjects were randomized into two groups; one group (*n* = 20) received rTMS for 10 days and the other group (*n* = 18) received propranolol for 30 days. This prescription included an initial dose of 10 mg three times a day (30 mg/day) if patients had no side effects, with an increase to 60 mg/day after 5 days and then to 90 mg/day after 10 days.

The Fahn–Tolosa–Marin (FTM) clinical scale, which consists of three subscales, was used to evaluate clinical effects. Part A evaluates tremor location/severity (amplitude), Part B assesses specific motor tasks (writing, spiral drawing, and pouring using dominant and nondominant hands), and Part C measures functional disability due to tremor (speaking, eating, drinking, hygiene, dressing, writing, working, and social activities of daily living) (Fahn et al., 1993). FTM scores range from 0 to 156, and the higher the score, the more severe the tremor. A neurologist (Doctor Yang) who was unaware of the intervention took measurements at baseline (before therapy) and days 5, 10, and 30 after the intervention. Throughout the entire intervention period, we monitored adverse events at every visit or upon patient report.

### 2.3 rTMS cerebellar stimulation

In the rTMS group, patients received 600 pulses of rTMS in each cerebellar hemisphere at a frequency of 1 Hz and an intensity of 90% of the resting motor threshold (RMT) for 10 consecutive days. There were 20 trains for each 1‐Hz rTMS, and each train was performed for 30 s and followed by a 5‐s break, and each treatment lasted approximately 20 min for every day. The RMT was measured based on the lowest stimulation intensity required to produce only five visible contractions out of 10 stimulations of a target muscle (the right abductor pollicis brevis muscle in our case). We employed rTMS with a figure‐eight coil connected to a Magstim Rapid^2^ System (The Magstim Company Limited, Whitland, UK). On each side of the cerebellum, the coil was positioned 3 cm lateral and 1 cm inferior to the inion, and the coil was placed tangent to the surface of the skull. We employed this position based on previous research showing that using this area can effectively stimulate the cerebellum (Hardwick et al., 2014).

### 2.4 Statistical analysis

All statistics were performed using IMB SPSS for Windows, Version 21.0 (Armonk, NY), and all graphs were generated using GraphPad Prism 8.0. Continuous variables were expressed as the mean ± SD or median (25th percentile, 75th percentile), whereas categorical variables were expressed as percentages (%). Continuous variables used student's *t*‐test (normal distribution) and Mann–Whitney *U* test (non‐Gaussian distribution), and categorical variables used *χ* ^2^ test. A significance level of 5% was assumed for this analysis. Statistical significance was defined as *p* < .05.

## 3 RESULTS

All 38 patients received their intended treatments. Twenty patients received rTMS of the bilateral cerebellum for 10 days, and all subjects tolerated rTMS sessions well without severe adverse effects (i.e., dizziness, headache, seizure, syncope). Eighteen patients received medical treatment with oral propranolol for 30 days. The initial dose was 30 mg/day, and the dose was increased to 60 mg/day after 5 days, then to 90 mg/day after 10 days, and continued thereafter for 20 days. None of the participants reported any adverse side effects (i.e., bradycardia, hypotension).

### 3.1 General clinical data

Of the 38 patients, 19 (50.0%) were female. Twenty‐two subjects (57.9%) had a positive family (first‐ or second‐degree relatives) history of ET. The mean age of the patients was 55.50 ± 13.55 years, and the mean duration of the tremor was 10.08 ± 8.30 years. All patients presented with hand tremor. Other locations of tremor were head (*n* = 14), leg (*n* = 1), trunk (*n* = 1), and face (*n* = 1). There was no significant difference in the general clinical data between the two groups, including sex, age, family history, tremor duration, and severity of tremor, on the baseline FTM scale (Table 1).

**TABLE 1:** Baseline characteristics of the participants

	rTMS (*n* = 20)	Propranolol (*n* = 18)	*p*
Number of female (%)	11 (55.0)	8 (44.4)	.516
Age (years)	56.40 ± 12.50	54.50 ± 14.94	.759
Duration of tremor (years)	11.20 ± 8.10	8.83 ± 8.60	.251
Positive family history (%)	12 (60.0)	10 (55.6)	.782
Head tremor (%)	9 (45.0)	5 (27.8)	.272
FTM score Part A	7.15 ± 2.92	6.72 ± 3.14	.576
FTM score Part B	11.75 ± 5.66	14.06 ± 6.60	.298
FTM score Part C	9.75 ± 3.61	9.83 ± 4.40	.906
FTM total score	28.65 ± 10.18	30.61 ± 12.97	.558

Abbreviations: FTM, Fahn–Tolosa–Marin clinical scale; rTMS, repetitive transcranial magnetic stimulation.

### 3.2 rTMS group

For the rTMS group, there was no significant effect in any clinical aspects of tremor: FTM score Part A, tremor severity (*p* = .242); FTM score Part B, specific motor tasks (*p* = .197); FTM score Part C, functional disability (*p* = .549); and FTM total score (*p* = .155) on day 5 compared with baseline, as shown in Table 2. Statistics indicated that compared with the scores at baseline, tremor severity (*p* = .006), specific motor tasks (*p* = .034), and FTM total score (*p* = .010) were significantly reduced on day 10 after rTMS and persisted until day 30 (tremor severity [*p* = .011], specific motor tasks [*p* = .039], and FTM total score [*p* = .013]). Functional disability did not differ at either time point (day 10 [*p* = .061], day 30 [*p* = .060]) (Table 2).

**TABLE 2:** Fahn–Tolosa–Marin (FTM) clinical scale of ET patients before and after cerebellar rTMS (mean ± SD)

	Baseline	Day 5	Day 10	Day 30
FTM Part A	7.15 ± 2.92	5.95 ± 2.44	4.55 ± 1.99	4.70 ± 2.11
FTM Part B	11.75 ± 5.66	10.20 ± 5.15	8.40 ± 4.77	8.60 ± 4.73
FTM Part C	9.75 ± 3.61	9.00 ± 3.43	7.50 ± 3.40	7.50 ± 3.63
FTM total	28.65 ± 10.84	25.15 ± 9.54	20.45 ± 9.01	20.80 ± 9.29

Abbreviation: rTMS, repetitive transcranial magnetic stimulation.

### 3.3 Propranolol group

No aspect of tremor improved significantly at day 5 and day 10 after application of propranolol, and there were no obvious differences for FTM subscales and total score, including tremor severity (day 5 [*p* = .641], day 10 [*p* = .279]), specific motor tasks (day 5 [*p* = .645], day 10 [*p* = .128]), functional disability (day 5 [*p* = .823], day 10 [*p* = .301]), and FTM Total score (day 5 [*p* = .669], day 10 [*p* = .189]), as shown in Table 3. On day 30 of oral propranolol treatment, all tremor parameters were significantly improved. For all scores, tremor severity (*p* = .024), specific motor tasks (*p* = .013), functional disability (*p* = .026), and FTM total score (*p* = .012) were significantly reduced compared with baseline (Table 3).

**TABLE 3:** Fahn–Tolosa–Marin (FTM) clinical scale of ET patients before and after oral propranolol (mean ± SD)

	Baseline	Day 5	Day 10	Day 30
FTM Part A	6.72 ± 3.14	6.33 ± 3.07	5.67 ± 2.95	4.50 ± 2.26
FTM Part B	14.06 ± 6.60	13.33 ± 6.58	11.39 ± 5.64	9.00 ± 5.29
FTM Part C	9.83 ± 4.40	9.67 ± 4.35	8.61 ± 4.06	6.66 ± 3.11
FTM total	30.61 ± 12.97	29.33 ± 12.79	25.67 ± 11.14	20.16 ± 9.39

### 3.4 Comparisons between two groups

**FIGURE 1:**
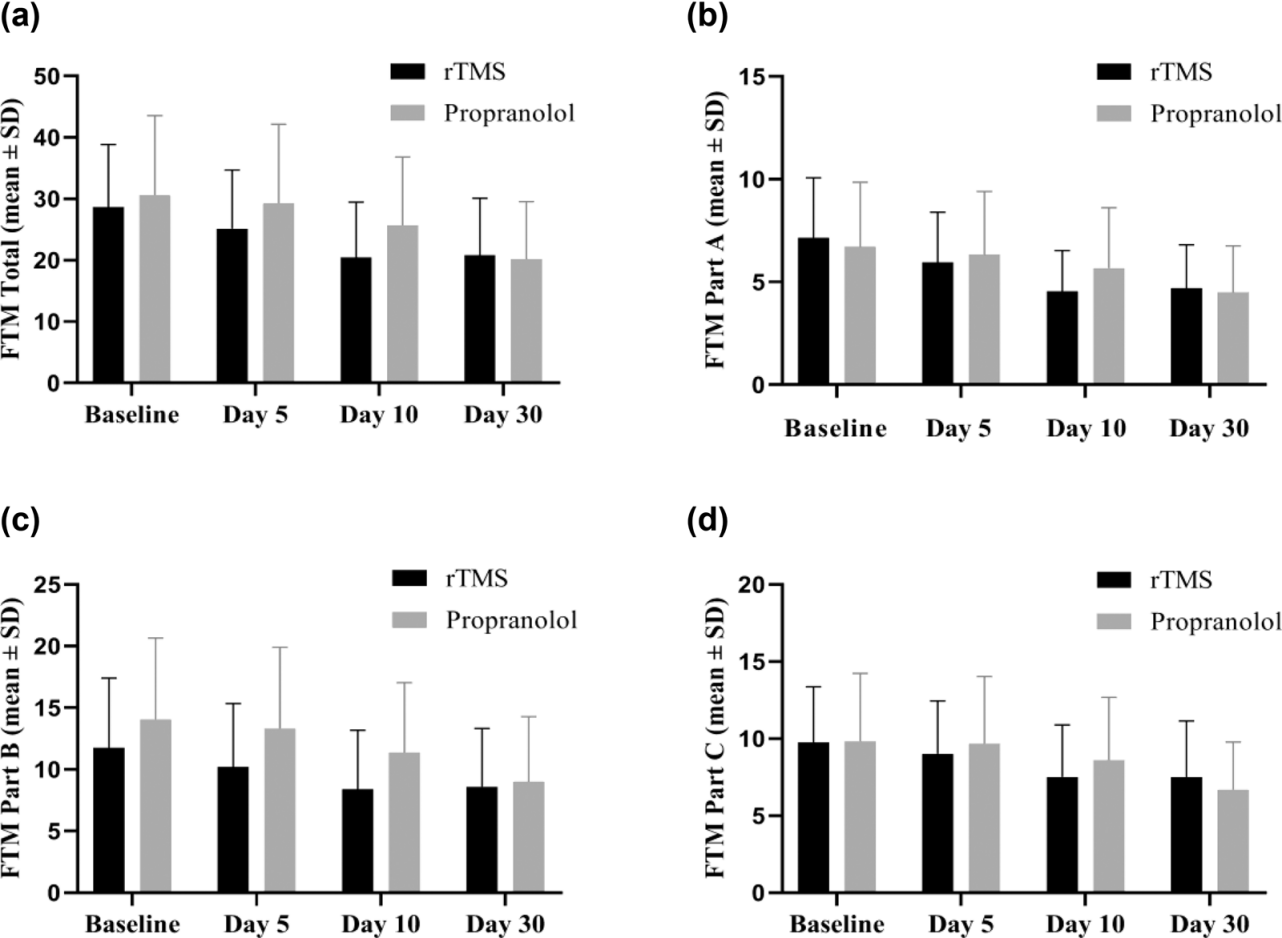
Comparison of Fahn–Tolosa–Marin clinical scale between repetitive transcranial magnetic stimulation group and propranolol group on baseline, day 5, day 10, and day 30. (a) FTM total score. (b) FTM score Part A, tremor severity. (c) FTM score Part B, specific motor tasks. (d) FTM score Part C, functional disability.

Comparisons between the two groups indicated that for the FTM total score, the treatment difference (rTMS effect‐propranolol effect) was not significant on day 5 (*p* = .198), day 10 (*p* = .147), or day 30 (*p* = .639) (Figure 1A). Additionally, treatment differences were not significant for FTM subscales, including tremor severity (day 5 [*p* = .836], day 10 [*p* = .375], and day 30 [*p* = .535]), performance of motor tasks (day 5 [*p* = .127], day 10 [*p* = .120], and day 30 [*p* = .736]), and functional disability (day 5 [*p* = .537], day 10 [*p* = .331], and day 30 [*p* = .517]) (Figure 1).

## 4 DISCUSSION

ET is one of the most common movement disorders, and multiple treatments are available (Sharma & Pandey, 2020). The choice of treatment depends on the anatomical site of tremor, tremor severity, functional disability, or other complications, and the most important consideration is the patients’ desire for treatment. In this study, we compared the efficacy of rTMS and propranolol in the treatment of ET. Our study observed significant and long‐lasting (20 days) improvement in FTM score Part A and Part B and total score in ET patients after 10 consecutive days of 1‐Hz rTMS sessions over cerebellar hemispheres, whereas FTM score Part C did not change at any time point. Part A and Part B were measured by neurological examination and evaluated for the severity of tremor performance in specific motor tasks, and Part C was measured through interviews and assessed for the impact of tremor on the daily lives of patients. Our results indicated that rTMS treatment reduced the severity of tremor but did not improve the function of patients’ daily lives. These findings are identical to the previous observation of Shin et al. (2019); the divergence of the subscores evaluated after rTMS treatment showed that a reduction in the severity of tremor, as appraised by neurological clinical examination, did not improve the daily functioning of ET patients. All of these results suggest that the reduction in tremor severity after rTMS treatment may not improve daily functioning in ET patients. A possible explanation for this result is that the severity of tremor was measured by neurological examination and evaluated for the severity of tremor performance in specific motor tasks. Functional disability due to tremor was measured through interviews, which have obvious subjectivity. In the future, we hope that more objective tools, such as accelerometers, will be used to evaluate the effectiveness of treatment; such instruments could potentially detect small changes in the severity of tremor.

In our study, we found that when patients took propranolol orally at a dose of 30 or 60 mg for the first 10 days, there was no significant change in any of the FTM subscores or the total score. We observed that there was a significant reduction in tremor severity and significant improvement in functional disability (including writing/drawing/pouring of water) in patients when the dose was increased to 90 mg and administered for another 20 days. According to the evidence‐based ET treatment guidelines from the American Academy of Neurology, class I evidence supports the successful use of propranolol in ET treatment (Zesiewicz et al., 2011). Previous studies have shown that the initial dose of short‐acting propranolol was 10 mg twice/thrice a day, gradually increased to 20–30 mg thrice daily, and the maintenance dose was 10–320 mg/day (Ferreira et al., 2019). Furthermore, published controlled trials have shown that propranolol at a dose of 120–240 mg/day has been suggested to elicit a clinically therapeutic effect, and the most common interview period ranged from 2 weeks to 1 month (Ferreira et al., 2019; Schneider & Deuschl, 2014; Sharma & Pandey, 2020; Zappia et al., 2013). Nevertheless, the clinical response to propranolol treatment was highly variable. Research has shown that compared with placebo, propranolol relieves clinical symptoms in approximately 40%–50% of patients, but some patients do not respond to the drug (Zhang et al., 2020). In addition, although side effects of propranolol could increase over time, treatments were generally well tolerated (Ondo, 2020). Bradycardia and bronchospasm were the most common side effects of propranolol in previous studies (Ferreira et al., 2019). In our study, to reduce serious drug side effects, low‐dose propranolol was chosen for ET patients, which could be one of the main reasons for the poor therapeutic effects on days 5 and 10.

Our research results also presented that there was no statistically significant difference in the therapeutic effect between rTMS and propranolol at any time point. In a recent single‐center, single‐blinded, randomized, sham‐controlled pilot study, all patients kept taking propranolol at their original dosage during all rTMS applications of the same protocol as our study, and they concluded that rTMS as an “add‐on” treatment had no obvious effect on ET patients who took propranolol (Shin et al., 2019). Moreover, no other similar studies have compared the effect of cerebellar low‐frequency rTMS with propranolol for ET patients. Although our result was negative, it is meaningful because this was the first study to compare the effects of rTMS and medication for ET patients.

Previous studies have shown that dysfunction of the cerebellum is crucial in the pathogenesis of ET. Using a combination of electromyography and fMRI, researchers have found increased tremor‐related activity in multiple regions of the bilateral cerebellum of ET patients (Maas et al., 2020). Based on this mechanism, the cerebellum is the most commonly used site in previous studies of NIBS treatment in ET patients. Combining our findings with previous reports, inhibitory stimulation of the cerebellar hemisphere was found to reduce the severity of tremor in ET patients with both short‐term and long‐lasting effects. An open‐label study evaluated the curative effect of inhibitory transcranial direct current stimulation (tDCS) applied to the cerebellar region for ET patients, and observed significant improvements in the severity of tremor and activities of daily living (Helvaci et al., 2016). Furthermore, in another double‐blind, crossover, and placebo‐controlled study, Gironell et al. (2014) also assessed the efficacy and safety of cerebellar tDCS for ET patients; nevertheless, they did not show any significant differences in tremor severity or daily living activities. Increasing evidence indicates that the cerebellum plays a key role in the pathophysiology of ET. These researchers found an increase in tremor‐related activity in multiple areas of the bilateral cerebellum by using a combined electromyography/functional MRI (fMRI) approach (Maas et al., 2020). Accordingly, based on this mechanism, the cerebellum has been the most popular site in previous studies of rTMS in ET patients. In a recent sham‐controlled pilot study, rTMS was also applied to the presupplementary motor area (pre‐SMA) to evaluate the therapeutic effect for ET (Badran et al., 2016). Badran et al. demonstrated that the FTM score was significantly lower than the baseline (26.11%) after 15‐day sessions of low‐frequency (1 Hz) rTMS administered to the pre‐SMA in patients with ET; however, sham rTMS also showed a reduction in FTM score (18.82%), and between‐group analysis demonstrated that scores barely reached statistical significance. However, during the 4‐ and 8‐week follow‐ups, only the active group maintained a significant decline compared to baseline. The above results indicate that differences in brain stimulation tools, sites, and paradigms used in NIBS research may produce different results. Future research on rTMS in the treatment of ET should focus on exploring the differences in the efficacy of different stimulation parameters in order to choose a more reasonable treatment method.

When interpreting our results, some limitations of our study should be taken into account. First, since we did not design a sham stimulation control during rTMS treatment, we cannot completely rule out the placebo effect on ET patients. In a double‐blind, sham‐controlled, crossover, add‐on clinical trial, patients were divided into two groups to receive rTMS and sham stimulation treatments and crossed over after a 2‐month washout period. In this study, one patient who received sham stimulation had at least a 70% reduction in the severity of tremor on day 5 compared with the baseline total FTM scores. They also found that compared with sham stimulation on day 5, 12, or 30, the total FTM scores in rTMS did not significantly improve (Olfati et al., 2020). A previous study showed that after treatment with placebo, more than half of the patients with tremor‐dominant Parkinson's disease had an amplitude of resting tremor that was reduced by at least 70% (Barbagallo et al., 2018). Similarly, there was also a significant placebo effect in the medical treatment of patients with ET (Ferreira et al., 2018). Future studies should recruit larger samples to systematically assess the placebo response. Second, during treatment with rTMS, we did not use any neuronavigation technique when positioning the stimulation site, so we cannot rule out the possibility that the coils were improperly positioned. Nevertheless, physicians usually use the inion as a surface marker in clinical treatment, and our study better reflects a real clinical practice situation; hence, this could be considered a strength of this study. Third, in this study, we used only clinical scales to analyze the severity of tremor without using more objective tools, such as accelerometers, which could possibly detect small changes in the severity of tremor. Since the score on the FTM scale was divided into four points ranging from 0 to 4 points, it could not accurately assess the exact change in tremor amplitude. Accelerometric measurement is likely to be a better tool for this type of research.

## 5 CONCLUSION

Our findings suggest that 1‐Hz cerebellar rTMS is a relatively safe and potentially effective noninvasive treatment technique for ET patients that can reduce the amplitude of tremor and improve the patient's specific motor tasks. Meanwhile, because of its advantages of convenience, effectiveness, and relatively cheap price, rTMS is easy to be accepted by patients. And the same effect could also be observed in treatment with propranolol. However, we did not find any differences between the two treatments. Future studies should include larger sample sizes of ET patients to explore other stimulation sites and parameter selection for rTMS and exploit more rigorous designs, such as sham stimulation, to address the placebo effect and other limitations.

## AUTHOR CONTRIBUTIONS

The work described here was done in mutual assistance with all the authors. Haining Li contributed to the research design and reviewed and edited the manuscript. Jiang Cheng collected references and revised the manuscript. Yue Lv completed the data collection, performed statistical analysis, and drafted the manuscript. Juan Yang and Mengran Wang conducted data collection. All the authors read and approved the final version of the manuscript.

## CONFLICT OF INTEREST STATEMENT

The authors declare no conflicts of interest.

### PEER REVIEW

The peer review history for this article is available at https://publons.com/publon/10.1002/brb3.2926.

## Data Availability

The datasets used and analyzed in the current study are available from the corresponding author on reasonable request.
